# Editorial: Social determinants of kidney health: a global perspective

**DOI:** 10.3389/fneph.2023.1260221

**Published:** 2023-08-14

**Authors:** Guillermo Garcia-Garcia, Keith C. Norris, Manisha Sahay, Ifeoma I. Ulasi

**Affiliations:** ^1^Department of Medical Clinics, University of Guadalajara Health Sciences Center, Guadalajara, Mexico; ^2^Department of Medicine, David Geffen School of Medicine, UCLA, Los Angeles, CA, United States; ^3^Department of Nephrology, Osmania Medical College and Hospital, Hyderabad, India; ^4^Department of Medicine, University of Nigeria College of Medicine, Enugu, Nigeria

**Keywords:** social determinants of health, chronic kidney disease, acute kidney injury, chronic kidney disease of nontraditional etiology (CKDnt), health disparities

*“It is not inequalities that kill people; it is those who are responsible for these inequalities that kill people.”*

*Vicente Navarro*

Social determinants of health (SDH) are the circumstances in which people are born, grow, work, live and age, including the broader set of forces and systems that influence the conditions of everyday life. They include a variety of factors such as education level, income, employment, housing, transportation, and access to healthy food, clean air and water, and health care services (**Figure 1**).

**Figure 1:**
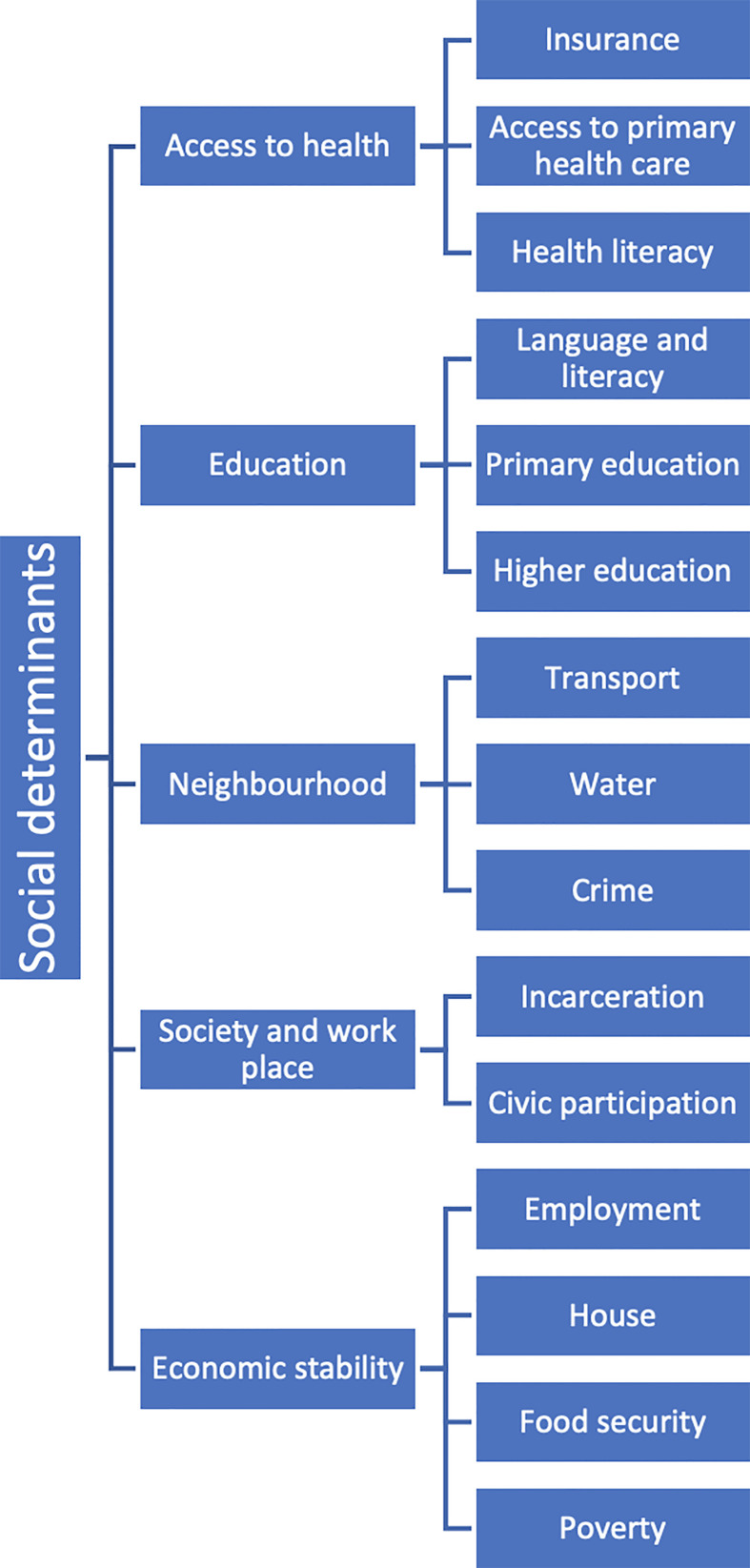
Social Determinants of Health.

There is ample evidence of the role played by the social determinants of health in the development of acute kidney injury (AKI) and chronic kidney disease (CKD), as well as their impact on its consequences and inequalities. The modification of the SDH aims to achieve greater equity in the distribution of resources and opportunities, to improve results in patients with kidney disease. Primary care physicians are in an ideal situation to identify the health and social needs of their patients and address them in their daily practice.

In this Research Topic, we present new studies and a review of the literature, which we hope will be useful to decision makers and health professionals, to understand the impact of social factors on kidney health and how this can influence the prevention and treatment of the actual disease.

The impact of socioeconomic status, education, gender and race on kidney health in India are evaluated by (Anandh et al.). The most important factor in inequalities in access to health is economic, especially in children, impacting on the morbidity and mortality of kidney patients. Gender disparities result in inequalities in access to health, greater vulnerability to disease, healthy behaviors, and biases in medical research. As elsewhere, access to transplantation is limited for women. Initiatives such as the Janani Shishu Suraksha Karyakaram and a more equitable distribution of deceased donor organs are some of the activities focused on reducing disparities in Indian women.

Globally, discrimination by race or ethnic groups results in adverse health outcomes. In India, the existence of a complex caste system causes inequality in access to health in the lower castes, especially in rural areas. To achieve universal health access various policies and programs in India have been implemented in the promotion of kidney health. For example, the implementation of the Medical Termination of Pregnancy Act has led to a drastic reduction in illegal abortions with a positive impact on the incidence of pregnancy-related AKI.

The influence of geographic location on the environment and its possible effect on kidney health is discussed by Aoun and Chelala. They provide evidence that CKD epidemiology, access to health care, and outcomes of CKD patients varies according to the geographical location where they reside. Living at high altitudes has been associated with proteinuria, hypertensives crisis, AKI and progression of CKD. Exposure to low temperatures increases the risk of hypertensive events and indirectly affects kidney function, whereas exposure to high temperatures increases the risk of dehydration and AKI. Seasonality and climate change are important emerging factors that impact kidney health and contribute to exacerbating kidney diseases such as lupus nephritis. The impact of occupation on kidney health of workers is analyzed based on social, temporality, and geographical factors. The exposure to nephrotoxins, infections, and pollutants as risk factor for developing kidney disease is well documented. Recently, a new type of occupational kidney disease, better known as CKD of non-traditional origin (CKDnT), has been described in agricultural workers exposed to contaminated water, like the case in Sri Lanka, or to heat stress and dehydration among agricultural workers in Central America. Similar types of occupational kidney disease have been reported elsewhere.

Hydrocarbon exposure has been linked to non-communicable diseases, including CKD, mostly as an occupational risk. In a case-control study, Okoye and Awunor evaluated the risk of presenting CKD by the environmental exposure to hydrocarbons in young Nigerians residing in contaminated areas in Nigeria’s Niger Delta region. Thirty-four (45.9%) of the cases were born and resided near to petrochemical plants, nearly 5 times that of the control group, suggesting a link between exposure to hydrocarbons and kidney disease. The findings emphasized the importance of residential and occupational history in evaluating of CKD patients.

Chronic kidney disease impacts the psychosocial, physical, and behavioral aspects of the patient, causing a poor self-rated health (SRH) assessment. In the analysis of the Longitudinal Ageing Study in India by Nayak et al., age ≥ 75 years, living in rural areas, and having more than one chronic disease increased the likelihood of poor SRH. Twenty-one percent of CKD patients did not receive health care, highlighting the need for a more accessible and affordable healthcare system.

Ruiz-Velazco et al., utilizing a qualitative, phenomenological-descriptive analysis, describe the complex relationship between the social and environmental determinants of health and the development of CKD of unknown etiology in infants residing in poor communities in the shores of Chapala Lake, Mexico. The study was conducted utilizing a narrative design. Living in poverty and having kidney disease results in greater impoverishment and lack of access to health care, especially for those without social security. People recognize the pollution of Lake Chapala, however interaction with the lake is indispensable since their everyday life is built around the lake. Late treatment results from a combination of poor early medical recognition, limited resources for timely laboratorial evaluation, and poor knowledge about kidney disease. The results raise the need for a transdisciplinary approach, evaluating people’s knowledge and how they experience kidney disease, elaborating holistic strategies on public health to improve outcomes in this population.

Finally, in a challenging paper, Kierans and Padilla-Altamira, argue that CKDnt can appear as an enigma, especially when viewed through an SDH framework that relies on a linear cause-effect analysis. They stress the need for new paradigms of understanding and action capable of complexity and mindful of context, social relations, and temporality. Collaboration between public health and anthropology is one place to start. We can understand emergent conditions on multiple scales by placing CKDnt in an SHD context and embracing its politics. The place to start is in local settings and with local populations. Engaging the community and health professionals lies at the center of this.

## Author contributions

GG: Writing – original draft. KN: Writing – original draft. MS: Writing – original draft. IU: Writing – original draft.

